# The effect of letrozole overlapped with gonadotropin on IVF outcomes in women with DOR or aged over 40 years old with repeated cycles

**DOI:** 10.1186/s13048-023-01273-4

**Published:** 2023-09-18

**Authors:** Xiaojia Li, Jingbo Chen, Yang Zhao, Fengyi He, Meijun Zeng, Guijun Guan, Xiaomiao Zhao

**Affiliations:** 1 Center for Reproductive Medicine, Department of Obstetrics and Gynecology, Sun Yat-sen Memorial Hospital, Sun Yat-sen University, Guangzhou, 510120 China; 2 Department of Reproductive Medicine, Guangdong Provincial People’s Hospital(Guangdong Academy of Medical Sciences), Southern Medical University, Guangzhou, 510080 China; 3 Department of Clinical Nutrition, Sun Yat-sen Memorial Hospital, Sun Yat-sen University, Guangzhou, 510120 China; 4 Key Laboratory of Exploration and Utilization of Aquatic Genetic Resources, Ministry of Education, Shanghai, 201306 China; 5 Shanghai Collaborative Innovation for Aquatic Animal Genetics and Breeding, College of Fisheries and Life Sciences, Shanghai Ocean University, Shanghai, China

**Keywords:** Aged, Diminished ovarian reserve, Letrozole, Repeated cycles

## Abstract

**Background:**

Evaluating the efficacy of letrozole overlapped with gonadotropin-modified letrozole protocol (mLP) for diminished ovarian reserve (DOR) or advanced-age women with repeated cycles.

**Methods:**

This is a retrospectively registered, paired-match study including 243 women with DOR and 249 women aged over 40 years old who received in vitro fertilization (IVF) treatment. 123 women received stimulation with mLP (mLP group). GnRH agonist (GnRH-a) long, GnRH antagonist (GnRH-anta), and mild stimulation protocol were used as controls with 123 women in each group. We further analyzed 50 of 123 patients in the mLP group who have experienced more than one failed cycles with other ovarian stimulation protocols (non-mLP group). Clinical pregnancy rate (CPR), cumulative clinical pregnancy rate (CCPR), and live birth rate (LBR) were main outcomes.

**Results:**

The CPR in the mLP group (38.46%) was significantly higher than mild stimulation (17.11%), but not significantly different from GnRH-a long (26.13%) and GnRH-anta (29.17%) group. The CCPR showed an increasing trend in the mLP group (33.33%) although without significance when compared with controls. The CCRP of GnRH-a long, GnRH-anta, mild stimulation group were 21.68%, 29.03%, and 13.04%, respectively. In women with repeated cycles, mLP achieved the higher available embryo rate (*P* < 0.05), the top-quality embryo rate, the CPR (*P* < 0.001), and the LBR (*P* < 0.001). Further study showed a positive correlation between testosterone and the number of oocytes retrieved in the mLP group (r = 0.395, *P* < 0.01).

**Conclusion:**

The mLP may be effective for aged or DOR women who have experienced previous cycle failure by improving the quality of embryos, the CPR, and the LBR. An increasing serum testosterone level may reflect follicular growth during ovarian stimulation.

**Supplementary Information:**

The online version contains supplementary material available at 10.1186/s13048-023-01273-4.

## Background

Diminished ovarian reserve (DOR) refers to women who have regular menses and normal response to ovarian stimulation but have unexpectedly lower fertility potential for their age, which implies early depletion of the actual primordial follicle pool [1]. Together with advanced age, an important factor affecting fertility, these make up two major challenges for assisted reproductive technology (ART). The gradual decline in the quality and quantity of ovarian oocytes are the major obstacles, leading to clinical pregnancy rates of only 10–15% among women over 40 years old [2]. Women with DOR or advanced age showed similar fertility characteristics, including decreased anti-Műllerian hormone (AMH), testosterone (T), and antral follicle count (AFC), corresponding to poor outcomes [3].

For years, numerous methods have been conducted to obtain better outcomes in women with advanced age or DOR. Increasing doses of gonadotropin (Gn), exogenous luteinizing hormone (LH) or growth hormone (GH) supplementation, and various kinds of controlled ovarian stimulation (COS) protocols were reported [4–8]. However, there is still no consensus due to the contradictory reports of comparable reproductive outcomes.

During ovarian stimulation, estrogen and androgen are important in the recruitment of primordial follicles and the promotion of follicular growth at the preantral and antral stages across different species [9]. Our previous study also suggested that basal total testosterone (TT) was a predictor for better outcomes of in vitro fertilization (IVF) [10], which was correlated with telomerase activity in human luteinized granulosa cells [11]. However, simply adding androgen has limited benefits due to the increase of plasma concentration throughout the entire body, but not locally in the ovaries [12]. Letrozole, a third-generation aromatase inhibitor widely used in ovarian stimulation by reducing the conversion of androgen to estrogen, consequently, results in a hypoestrogenic state which increases GnRH and pituitary follicle-stimulating hormone (FSH) release for ovulation. It promotes the growth of more follicles by raising the threshold of follicular growth and prolonging the threshold window [13]. To promote the maturation of follicles as much as possible, we combined 5 mg/day of letrozole from day 2 of the menstrual cycle for five days with 225–300 IU/d of Gn, termed the modified letrozole protocol (mLP).

## Methods

### Study Period and Population

This study was retrospectively registered. 243 women with DOR and 249 women over 40 years old who underwent IVF or ICSI at the Reproductive Medicine Center of Sun Yat-sen Memorial Hospital, Sun Yat-sen University from January 2016 to July 2020 were included in this study. In this study, we defined DOR as AMH < 1.2 ng/mL or AFC < 5 in women under 40 years old, according to Bologna and Poseidon criteria for POR and DOR [14, 15]. Patients with a history of recurrent pregnancy loss, polycystic ovarian syndrome, hyperprolactinemia, diabetes mellitus, uncontrolled thyroid dysfunction, untreated submucous myoma, endometrial polyp larger than 1 cm, or congenital uterine malformations were excluded. The study was approved by the Medical Ethics Committee of the Sun Yat-sen Memorial Hospital of Sun Yat-sen University (Ethical approval: 2019-KY-067). All participants received adequate counseling regarding the stimulation regimens and signed informed consent forms before stimulation.

### Study Design

#### Ovarian stimulation and follicle monitoring

MLP was the experimental protocol of COS, while standard luteal GnRH agonist long protocol (GnRH-a long protocol), GnRH antagonist protocol (GnRH-anta protocol), and mild ovarian stimulation protocol were paired-matched with age and AMH as controls, where there were 123 cases in each group. Detailed information on the protocols was shown in Supplemental Fig. 1.

(1) Modified letrozole protocol (mLP): Patients who received the mLP protocol were treated with letrozole (Femara; Novartis Pharma AG, Basel, Switzerland) at an oral dose of 5 mg/day which was initiated on day 2 of the menstrual cycle for five days to day 6 and overlapped with FSH/human menopausal gonadotropin (HMG) at a dose of 225–300 IU/day (Lishenbao and Lebaode, Livzon, Zhuhai, China; Menopur, Ferring, German; Gonal-f, MerckSerono, Switzerland) subcutaneously or intramuscularly from day 5 of the menstrual cycle. GnRH antagonist (Ganirelix, Vetter Pharma-Fertigung GmbH & Co. KG, German; Cetrorelix, Merck Serono, Geneva, Switzerland) was initiated in the following situations to prevent premature ovulation when the LH level reached above 10 IU/day or the diameter of follicles were larger than 14 mm.

(2) GnRH-a long protocol: Patients underwent pituitary down-regulation with GnRH agonist (Triptorelin Acetate, Ipsen Pharma Biotech, France) at the mid-luteal phase of the menstrual cycle (days 18–20). When the concentration of serum E2 was less than 50 ng/L and the endometrial thickness was less than 5 mm with an absence of 10 mm large follicles in both ovaries by transvaginal ultrasound, FSH/HMG was initiated at a dose of 225–300 IU/day until the trigger day.

(3) GnRH-anta protocol: Patients were commenced on FSH/HMG at 225–300 IU/day from day 2 of the menstrual cycle and the antagonist was administered at a dose of 0.25 mg/day from the 6th day of Gn stimulation (fixed protocol) or when the diameter of follicles was larger than 14 mm (flexible protocol) until the trigger day.

(4) Mild stimulation protocol: Patients received 5 mg/day letrozole or 50–100 mg clomiphene from day 2 to 3 of the menstrual cycle for 5 days, then in combination with 75–150 IU HMG per day, or directly with 75–150 IU/day HMG from day 2 or 3 of the menstrual cycle to the trigger day.

All patients underwent the measurement of endometrial thickness and follicle counting with transvaginal ultrasound as required. Blood samples were collected on days 2–4 of the menstrual cycle for basal serum hormone levels and at every visit during ovarian stimulation for FSH, LH, E2, and T. All serum hormones were measured using Access 2 chemiluminescence immunoassays (Beckman, Chaska, MN, USA) according to the manufacturer’s protocols.

#### Oocyte retrieval, embryo culture, grading, and transfer

When the diameter of dominant follicles reached 18 mm, an initial individualized dose (6000–10,000 IU) of human chorionic gonadotrophin (Pregnyl, Organon, Oss, Netherlands) or an additional 0.1 mg GnRH-a (dual trigger) was administered to trigger follicle maturation. Then, oocyte retrieval was performed 34–36 h later, guided by a transvaginal ultrasound scan. IVF or ICSI was performed according to the laboratory’s routine insemination procedures. Fresh embryo transfer (ET) was performed with 3-day cleaved embryos or 5-day blastocysts as the priority. The luteal phase was supported with daily administration of vaginal progesterone for 17 days starting on the day of oocyte retrieval, and if pregnancy occurred, the progesterone was continued for another 8 weeks. Serum levels of β-hCG and progesterone were measured 14 ± 3 days after embryo transformation. Clinical pregnancy was confirmed through ultrasonic observation of the intrauterine gestation sac at 3 weeks after a positive serum hCG test. Additionally, pregnancy and delivery status were followed up.

For women who underwent delayed embryo transfer, 3-day or 5-day embryos were cryopreserved, and frozen-thawed embryo transfer would be performed after proper endometrial preparation with oral estradiol valerate (Progynova, Bayer, Germany) in a hormone replacement therapy (HRT) cycle. Then, intramuscular progesterone was added at a dose of 60 mg/day when the endometrial thickness reached more than 8 mm. 3-day or 5-day frozen embryos were thawed and transferred on day 4 or 6 of the progesterone regimen, respectively. Luteal-phase support and further treatment were similar to fresh embryo transfer.

#### ART outcomes

The ART outcomes were compared among these four groups. The primary outcomes were clinical pregnancy rate (CPR) and cumulative pregnancy rate (CCPR) while secondary outcomes included number of oocytes received, follicular output rate (FOR), top-quality embryo rate, and miscarriage rate. Among these, CPR was defined as gestational sac and original fetal heart beat on ultrasonography on day 30 after fresh embryo transfer and the first frozen-thawed embryo transfer (FET) cycle for those who canceled fresh ET. CCPR was defined as the rate of clinical pregnancy in fresh and/or subsequent FET cycles in women who had available embryos. FOR was a parameter used to evaluate the responsiveness of antral follicles to exogenous hormones, which was defined as the ratio between the number of pre-ovulatory follicles (measuring 16–22 mm in diameter in both ovaries) on the trigger day and AFC [16].

#### Repeated cycles analysis between mLP and non-mLP groups

To validate the effectiveness of the mLP protocol in repeated cycles, we further analyzed 50 of 123 patients in this group (mLP group) who have experienced more than one failed cycles with other ovarian stimulation protocols (non-mLP group). Protocols used in previous cycles were listed in Table S1. Parameters of reproductive outcomes were analyzed to evaluate the effectiveness of the mLP protocol. The primary outcomes were CPR and live birth rate (LBR). The secondary outcomes included available embryo rate, top-quality embryo rate, and miscarriage rate.

### Statistical analysis

One-to-one case matching was performed using age and serum AMH level as the criteria. Continuous data of normal distribution were presented as mean ± standard deviation (Mean ± SD) and those not in accord were showed as median (25th percentile to 75th percentile). Qualitative variables were displayed in rate (number). The frequency distribution among different groups was assessed using ANOVA analysis, and the Student-Newman-Keuls method was utilized in paired comparisons when the *P* value of the ANOVA test was less than 0.05. The Kruskal-Wallis test was recommended for data that do not meet with normal distribution. For categorical variables, Pearson’s chi-square test or Fisher’s exact probability test was used. Correlations between parameters were analyzed by Spearman’s rank test. All results were statistically analyzed by SPSS version 25.0 (SPSS Inc., Chicago, IL, USA). *P* < 0.05 was considered statistically significant.

## Results

### Basic characteristics of subjects

The basic characteristics of subjects were summarized in Table 1 and Supplemental Fig. 2. After one-to-one case matching, the distribution of age and serum AMH level was similar in the four groups. Patients who received mLP had fewer AFC than the GnRH-anta group, longer infertile duration though without significance, and a higher proportion of primary infertility compared with the GnRH-a long protocol, as well as experiencing more cycles than the GnRH-a long and GnRH-anta groups. Other parameters including BMI and baseline hormonal levels were comparable among groups.

**Table 1:** Basic characteristic of patients, cycle parameters and reproductive outcomes of different COS protocols

Group	MLP group	GnRH-a long group	GnRH-anta group	Mild stimulation group	*P*
**Basic characteristic of patients**					
Number	123	123	123	123	
Cycle	2.02 ± 0.13^bc^	1.33 ± 0.06^ad^	1.54 ± 0.07^ad^	1.89 ± 0.07^bc^	< 0.01
Age (y)	37.76 ± 0.44	38.74 ± 0.41	38.63 ± 0.34	38.15 ± 0.42	0.298
BMI (kg/m^2^)	21.69 ± 0.28	22.36 ± 0.34	22.34 ± 0.26	22.26 ± 0.14	0.109
AMH (ng/ml)	1.14 ± 0.09	1.24 ± 0.08	1.18 ± 0.10	1.03 ± 0.09	0.358
AFC	5.02 ± 0.22^c^	5.63 ± 0.24	6.04 ± 0.30^ad^	5.02 ± 0.28^c^	0.012
Infertile duration (y)	5.33 ± 0.42	4.46 ± 0.35	4.99 ± 0.37	4.42 ± 0.38	0.262
Primary infertile (%)	39.02(48/123) ^b^	18.70(23/123) ^a^	28.46(35/123)	26.02(32/123)	0.005
**Basic hormone level**					
PRL_B_ (µg/L)	12.72(9.30–17.20)	11.43(8.42–15.07)	11.13(8.60-14.72)	11.38(8.53–15.35)	0.260
FSH_B_ (IU/L)	8.76(6.75–12.94)	8.65(7.28–11.12)	9.59(6.97–12.86)	9.70(7.27–12.54)	0.406
LH_B_ (IU/L)	3.95(2.84–5.25)	3.63(2.72–4.92)	3.76(2.93–4.98)	4.22(2.91–5.82)	0.155
E2_B_ (ng/L)	44.50(28.00-63.25)	38.00(27.50–58.00)	43.00(26.75–62.25)	44.50(28.00-59.25)	0.650
T_B_ (nmol/L)	1.10(0.63–1.72)	1.00(0.69–1.46)	1.23(0.68–1.55)	1.12(0.70–1.72)	0.410
**Cycle parameters**					
Days of Gn stimulation (d)	8.02 ± 0.23^bc^	12.57 ± 0.17^acd^	8.78 ± 0.19^abd^	7.48 ± 0.26^abc^	< 0.01
Total Gn dose (IU)	2338.21 ± 71.30^bd^	3393.21 ± 73.21^acd^	2243.09 ± 64.71^bd^	1145.90 ± 48.09^abc^	< 0.01
**Trigger day**					
FSH (IU/L)	30.85(24.71–37.07)^bcd^	23.25(19.58–29.01)^ad^	21.58(18.67–27.42)^ad^	16.41(13.70-22.39)^abc^	< 0.01
LH (IU/L)	3.98(2.34–6.45)^bd^	1.21(0.93–1.65)^acd^	3.09(2.01–4.87)^bd^	5.76(3.03–11.32)^abc^	< 0.01
E2 (ng/L)	538.00(317.00-1039.75)^bc^	1407.00(861.00-2471.00)^ad^	1068.00(665.50–1797.00)^ad^	743.00(429.00-1182.00)^bc^	< 0.01
P (µg/L)	0.80(0.59–1.22)	0.96(0.66–1.26)^d^	0.93(0.63–1.28)	0.75(0.52–1.09)^b^	0.044
T (nmol/L)	2.12(1.36–2.84)^c^	1.90(1.67–2.92)	1.59(1.15–1.82)^a^	-	0.018
Number of follicles ≥ 18 mm	1.92 ± 0.11^ cd^	2.10 ± 0.13^d^	1.43 ± 0.11^ab^	1.08 ± 0.11^ab^	< 0.01
Follicle output rate (%)	37.58(236/628)^cd^	36.71(254/692)^cd^	23.42(174/743)^ab^	20.87(129/618)^b^	< 0.01
Endometrial thickness (mm)	9.44 ± 0.21^bcd^	11.39 ± 0.31^ad^	10.67 ± 0.25^ad^	8.35 ± 0.28^abc^	< 0.01
**IVF laboratory parameters**					
Number of oocytes retrieved	4.46 ± 0.26 ^bd^	6.71 ± 0.42^acd^	4.64 ± 0.31 ^bd^	2. 54 ± 0.21 ^abc^	< 0.01
MII oocytes retrieved	3.66 ± 0.23 ^bd^	5.65 ± 0.35^acd^	3.96 ± 0.28 ^bd^	2.12 ± 0.17^abc^	< 0.01
Available embryos	1.97 ± 0.13^bd^	2.52 ± 0.14^acd^	2.10 ± 0.13^bd^	1.34 ± 0.11 ^abc^	< 0.01
Top-quality embryo rate (%)	52.94(126/238) ^d^	58.77(181/308) ^d^	50.00(125/250)	38.61(61/158) ^ab^	< 0.01
**Pregnancy outcomes**					
Embryo implantation rate (%)	23.14(28/121)	16.47(28/170)	17.05(22/129)	13.56(8/59)	0.347
Clinical pregnancy rate (%)	38.46(35/91) ^d^	26.13(29/111)	29.17(30/103)	17.11(13/76) ^a^	0.022
Miscarriage rate (%)	11.54(3/26)	11.54(3/26)	25.00(5/20)	25.00(2/8)	0.528
Cumulative clinical pregnancy rate (%)	33.33(40/120) ^d^	21.68(31/143)	29.03(36/124)	13.04(15/115) ^a^	0.024

*Note*: MLP: modified letrozole protocol; BMI: body mass index; AMH: anti mullerian hormone; AFC: antral follicle count; PRL: Prolactin; FSH: follicle stimulating hormone; LH: luteinizing hormone; E2: estradiol; T: testosterone; P: Progesterone; Gn: gonadotropin; B in subscript means basic hormone level

^a^*P* < 0.05 when compared with modified letrozole group;

^b^*P* < 0.05 when compared with GnRH-a long protocol;

^c^*P* < 0.05 when compared with GnRH-ant protocol;

^d^*P* < 0.05 when compared with mild stimulation protocol

### Cycle characteristics and Reproductive Outcomes of different COS protocols

The cycle parameters and reproductive outcomes of the four different COS protocols were illustrated in Table 1. Patients in the GnRH-a long group were treated for the longest days and received the largest dose of Gn whereas the mild stimulation protocol received the least dose (*P <* 0.01). When it came to the trigger day, the FSH level in the mLP group was significantly higher than other groups, with a lower E2 level than GnRH-a long and GnRH-anta group and a higher T level than the GnRH-anta group. MLP group achieved 37.58% follicle output rate, which was comparable to the GnRH-a long group and was higher than that of the GnRH-anta group or mild stimulation group. The numbers of women who canceled oocyte pick-up in the four groups were three, one, two, and 10, respectively. Although GnRH-a long protocol reported the highest number of total and MII oocytes retrieved, the top-quality embryo rate of the mLP group, GnRH-a long group, and GnRH-anta group were comparable and higher than that of the mild stimulation group. As expected, the GnRH-a long group had the highest fresh embryo transfer rate. As for CPR, the mLP group showed a much higher trend (38.46%) compared with the mild stimulation group (17.11%) with statistical significance (*P* = 0.022), but the CPR was comparable with the GnRH-a long group (26.13%) and GnRH-anta group (29.17%). Similar tendencies of CCPR were shown in the four groups with statistical significance (*P* = 0.024), 33.33% for the mLP group, 21.68% for the GnRH-a long group, and 13.04% for the mild stimulation group **(**Table 1**)**.

### Cycle characteristics and Reproductive Outcomes of repeated cycles between MLP and Non-mLP groups

The cycle parameters and reproductive outcomes of repeated cycles between mLP and non-mLP groups were demonstrated in Table 2. By overlapping letrozole and 225–300 IU/d Gn, both FSH (33.16 IU/L vs. 23.69 IU/L, *P* < 0.01) and LH level (3.92 IU/L vs. 2.46 IU/L, *P* = 0.039) were increased with a significantly lower E2 level (436.50 ng/L vs. 1179.00 ng/L, *P* < 0.01) during the trigger day. The number of follicles larger than 18 mm on the trigger day showed an increasing trend in the mLP group, though without statistical significance. The number of oocytes and MII oocytes retrieved were similar between the two groups. Although previous cycles owned higher total and normal fertilization rates, the numbers of cleaved embryos were comparable. Moreover, in the mLP cycles, the rate of available embryos increased significantly (62.91% vs. 50.35%, *P* = 0.03) together with a relatively higher top-quality embryo rate (47.38% vs. 38.89%, *P* = 0.075). Although the mLP cycles showed significantly thinner endometrium (9.73 ± 0.36 vs. 10.35 ± 0.40, *P* = 0.034), the CPR (37.21% vs. 6.06%, *P* < 0.001) and the LBR (30.23% vs. 0, *P* < 0.001) both significantly increased, accompanied by the lower miscarriage rate (18.75% vs. 75.00%, *P* = 0.061), implying potentially higher-quality embryos can obtain better clinical outcomes (Table 2).

**Table 2:** Cycle parameters and reproductive outcomes of repeated cycles between mLP and non-mLP groups

Group	MLP group	Non-mLP group	*P*
Number	50	50	
**Cycle parameters**			
Total Gn dose (IU)	2443.00 ± 97.09	2521.00 ± 157.83	0.548
Days of Gn stimulation (days)	8.30 ± 0.32	10.26 ± 0.44	< 0.01
**Features of trigger day**			
FSH (IU/L)	33.16(24.84–39.92)	23.69(10.04–30.66)	< 0.01
LH (IU/L)	3.92(2.34–5.48)	2.46(1.45–6.38)	0.039
E_2_ (ng/L)	436.50(278.25–819.50)	1179.00(595.50-1750.75)	< 0.01
Number of follicles ≥ 18 mm	1.80 ± 0.17	1.50 ± 0.17	0.133
Endometrial thickness (mm)	9.73 ± 0.36	10.35 ± 0.40	0.034
**IVF laboratory parameters**			
Number of oocytes retrieved	4.16 ± 0.38	4.88 ± 0.70	0.860
Number of MII oocytes	3.42 ± 2.47	3.56 ± 2.34	0.724
MII oocytes retrieved rate (%)	82.21(171/208)	82.03(178/217)	0.961
Number of fertilized oocytes	2.70 ± 2.24	3.30 ± 2.27	0.092
Fertilization rate (%)	64.90(135/208)	76.04(165/217)	0.012
Normal fertilization rate (%)	70.37(95/135)	84.85(140/165)	< 0.01
Number of cleaved embryos	3.04 ± 2.48	3.22 ± 2.17	0.617
Available embryos rate (%)	62.91(95/151)	50.35(72/143)	0.03
Top-quality embryo rate (%)	47.38(45/95)	38.89(28/72)	0.075
**Pregnancy outcomes**			
Clinical pregnancy rate (%)	37.21 (16/43)	6.06 (4/66)	< 0.001
Miscarriage rate (%)	18.75 (3/16)	75.00 (3/4)	0.061
Live birth rate (%)	30.23 (13/43)	0	< 0.001

*Note*: MLP: modified letrozole protocol; Gn: gonadotropin; FSH: follicle stimulating hormone; LH: luteinizing hormone; E2: estradiol; T: testosterone; MII oocytes: metaphase II oocytes

### Correlation between Follicular Growth and Hormonal Level

**Fig. 1:**
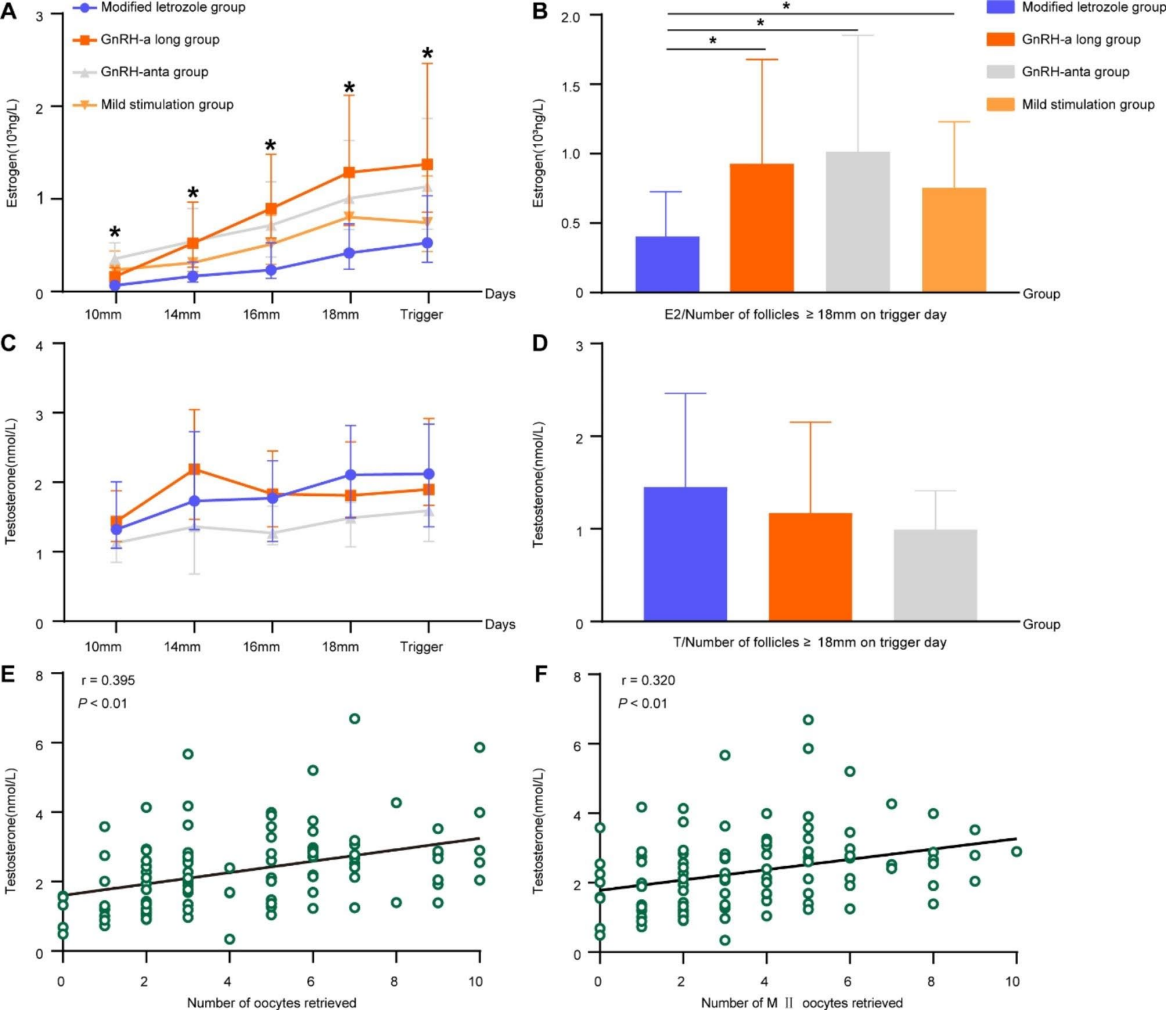
Estrogen and testosterone levels during ovarian stimulation in different groups. (**A-B**) Estrogen level on the day when follicles of different diameters first showed up and estrogen levels when follicles over 18 mm in diameter were present on the trigger day. Data were shown as mean ± standard deviation. * in Fig. 1A represents *P* < 0.05 among the modified letrozole group and others. (**C-D**) Testosterone levels on the day when follicles of different diameters first showed up and testosterone levels when follicles over 18 mm in diameter were present on the trigger day. Data were shown as mean ± standard deviation. * *P* < 0.05 between or among groups. (**E-F**) Correlation analysis between number of oocytes retrieved or number of MII oocytes retrieved and testosterone levels on the trigger day in the modified letrozole group

During ovarian stimulation, not only E2 but also testosterone level was found to show a rising trend with follicular growth. To investigate the relationship between follicular development and hormonal levels, we analyzed serum E2 and T levels when different diameters of follicles first showed up and found that the E2 level in the mLP group was significantly lower than the other three groups from the time large follicles of 10 mm in diameter first appeared, and this trend continued until the trigger day **(**Fig. 1A**)**. The ratio of E2 level to the number of follicles ≥ 18 mm on the trigger day was significantly lower in the mLP group (*P* < 0.05) **(**Fig. 1B**)**. Because only a few subjects in the mild stimulation group measured T levels during every visit for follicular monitoring, only three groups were compared for changes in T levels. When the biggest follicle reached 18 mm or larger, T levels showed an increasing trend in the mLP group in comparison with the two other groups, albeit without significance **(**Fig. 1C**)**. Additionally, the ratio of T level to the number of follicles ≥ 18 mm on the trigger day was also the highest, but without a statistical difference **(**Fig. 1D**)**. The number of oocytes retrieved (*r* = 0.3952; *P* < 0.01) and MII oocytes (*r* = 0.3201; *P* < 0.01) both had a positive correlation with T levels on the trigger day in the mLP group, indicating the potential role of T levels in predicting follicular maturation **(**Fig. 1E F**)**.

## Discussion

In this study, we aimed to evaluate the effectiveness of mLP for women with DOR or advanced age when undergoing controlled ovarian stimulation. Although AFC and basic hormonal levels were similar among groups, women of the mLP group had the most cycles ever experienced and the highest proportion of primary infertility but received similar clinical outcomes compared with traditional ovarian stimulation protocols. Furthermore, the mLP protocol also showed potential benefits for aged or DOR women who have previously experienced failed cycles by improving the quality of embryos, the CPR, and the LBR.

Either the GnRH-a long protocol or GnRH-anta protocol was usually utilized in routine practice at our IVF center for women with relatively better ovarian function among those with DOR or aged over 40 years old. For those who failed to achieve pregnancy, the mild ovarian stimulation protocol was a choice. When limited benefits were achieved using conventional protocols, mLP would be recommended. Therefore, it was shown that the AFC relatively appeared to be the best in the GnRH-anta protocol, then the GnRH-a long protocol, and the worst in the mLP and mild ovarian stimulation protocol, although pairing has been performed for age and AMH. Additionally, the number of IVF cycles experienced and the proportion of primary infertility were both higher in the mLP group, indicating that these patients have spent much time attempting to achieve pregnancy but failed to do so. However, even in such disadvantageous conditions, patients who underwent mLP could achieve comparable or even a rising tendency of reproductive outcomes compared with other groups, which was promising.

Ovarian stimulation protocols for women of advanced aged or DOR is still controversial. According to the Poseidon criteria, the GnRH-anta protocol or GnRH-a long protocol, in addition to a double trigger of hCG and GnRH-a, were relatively recommended among women with POR [15]. Huang et al. reported a CPR of 36.0% for women with POR younger than 35 years old with the GnRH-a long protocol and 26.2% with the GnRH-anta protocol; and 29.8% and 25.5% when it came to women aged 35 to 39 years old, and 11.4% and 8.4% among women aged 40 years old and above, respectively [17]. In our study, the CPR was 38.46% among women of advanced age or those with DOR treated with mLP, which was higher than the other three protocols, although without a statistical difference when compared with the GnRH-a long and GnRH-anta groups. Similar to Huang’s study, being one of the most commonly used and classic ovarian stimulation protocols, GnRH-a long group showed superior endometrial thickness, number of oocytes retrieved, and available embryos with the longest days of stimulation and the largest dose of Gn. Interestingly, when it comes to the trigger day, women of the mLP group reported the highest FSH level, followed by the GnRH-a long and GnRH-anta group, and the mild stimulation group was the lowest, which might be a result of the improvement of FSH sensitivity by pre-treatment with letrozole. When CCPR was calculated, the mLP group also showed an increasing trend, though without significance.

We also found a unique benefit of the mLP protocol for women with DOR or those over 40 years old with previous cycle failures by significantly increasing the available embryo rate and clinical pregnancy rate with even less Gn stimulation, and the CPR was 37.21% (12 of 33 women achieved pregnancy during fresh ET and 4 of 10 women who canceled fresh ET but received frozen ET achieved pregnancy), which was superior than the 22.58% of CPR reported by Chen et al. among infertile women of advanced age who failed to achieve pregnancy during their first IVF/ICSI cycle with the GnRH-a long protocol [18]. Similar results were also shown in all cases, which suggested that mLP may improve the quality of oocytes or endometrial receptivity, but not recruitment.

Currently, it is of consensus that sufficient androgenic action through the AR was necessary for normal follicular development and function. A critical balance exists between the essentiality of androgens in normal follicular development and their detrimental effects in hyper-androgenic conditions that affect female fertility [19]. What’s more, our previous study demonstrated that AMH and aromatase were discovered not only in ovaries but also in the central nervous system, and TGF-β signaling may be involved in the process by regulating the synthesis and release of FSH and LH [20–22]. Letrozole, which acts as an aromatase inhibitor, could reduce the conversion of androgen to estrogen. According to the literature, exogenous or endogenous testosterone could protect the genomic instability of embryos which was related to its anti-inflammatory properties [23]. Together with a relatively higher T level but lower E2 level in the mLP group during stimulation, androgen was confirmed to play a special role in follicular development and embryo implantation, which was also reported by other studies [24]. Therefore, we hypothesized that letrozole may play a role in follicular development through the potential mechanism on the hypothalamic-pituitary-ovarian (HPO) axis to increase endogenous androgen levels. We modified the letrozole protocol to utilize pre-treatment with letrozole from day 2 of the menstrual cycle for five days in addition to overlapping with a high dose of gonadotrophin. Pre-treatment with letrozole before gonadotrophin administration was assumed to be necessary to prolong the effect of modulation from the central nervous system to the ovaries via androgen signaling [24]. The addition of gonadotrophin at day 5 of the menstrual cycle within the FSH threshold window of AFC recruitment was another vital step to stimulate as many follicles as possible. Interestingly, with the lowest E2 level in the mLP group, the T level was higher than other groups, though without significance. Further analysis found a positive correlation between the number of retrieved oocytes and testosterone. These findings suggested that not only estrogen but also androgen could reflect follicular development. This is in agreement with a lower peak value of E2 present in patients with breast cancer with the use of letrozole in ovarian fertility preservation, although this point was not explicitly raised [25].

The study creatively proposed a novel COS protocol—mLP protocol, which was effective for improving the reproductive outcomes of DOR and advanced age populations, especially those who have previously experienced failed IVF/ICSI cycles. It provides those poor ovarian response women with a new option for ART treatment. However, the current study also has several limitations. It is a retrospective study that the patient allocation to the four COS protocols was not random. Although we have already paired for age and AMH, the presence of biases cannot be excluded. To avoid these biases of this retrospective study, a prospective multi-centered randomized clinical trial including seven IVF centers in different areas of China has been initiated with the modified letrozole protocol as the case group and the GnRH-anta protocol as the control group to further validate the efficacy of the novel protocol (Registration number: ChiCTR2000029272).

## Conclusions

The mLP may be effective for aged or DOR women who have previously experienced failed cycles by improving the quality of embryos, the CPR, and the LBR. An increasing serum testosterone level may play a role and reflect follicular growth during ovarian stimulation.

### Electronic supplementary material

**Supplementary Material 1**: Table 1 Ovarian stimulation protocols used in previous cycles of the modified letrozole groupSupplementary

**Supplementary Material 2**: Figure 1 Treatment scheme for four ovarian stimulation protocols. (A) Modified letrozole protocol. (B) GnRH agonist long protocol. (C) GnRH antagonist protocol. (D) Mild stimulation protocol

**Supplementary Material 3**: Figure 2 (A-D) Distribution of age, cycle, serum AMH level, and antral follicle count in different groups. G1: Modified letrozole group. G2: GnRH agonist long group. G3: GnRH antagonist group. G4: Mild stimulation group

## Data Availability

The raw data supporting the conclusions of this article will be made available by the authors, without undue reservation.

## Abbreviations

mLP: modified letrozole protocol
DOR: diminished ovarian reserve
IVF: in vitro fertilization
GnRH-a: GnRH agonist
GnRH-anta: GnRH antagonist
CPR: clinical pregnancy rate
CCPR: cumulative clinical pregnancy rate
LBR: live birth rate
ART: assisted reproductive technology
AMH: anti-Műllerian hormone
T: testosterone
AFC: antral follicle count
Gn: gonadotropin
LH: luteinizing hormone
GH: growth hormone
COS: controlled ovarian stimulation
FSH: follicle-stimulating hormone
HMG: human menopausal gonadotropin
ET: embryo transfer
HRT: hormone replacement therapy
FOR: follicular output rate
FET: frozen-thawed embryo transfer

## References

[1] Medicine PCotASfR (2015). Testing and interpreting measures of ovarian reserve: a committee opinion. Fertil Steril.

[2] Broekmans F, Kwee J, Hendriks D, Mol B, Lambalk C (2006). A systematic review of tests predicting ovarian reserve and IVF outcome. Hum Reprod Update.

[3] Gleicher N, Kim A, Weghofer A (2013). Hypoandrogenism in association with diminished functional ovarian reserve. Hum Reprod.

[4] D L, P D, E V, A A. Different ovarian stimulation protocols for women with diminished ovarian reserve. J Assist Reprod Genet. 2007; 24(12): 597–611. 10.1007/s10815-007-9181-2 PMC3455002 18034299

[5] Liu C, Jiang H, Zhang W, Yin H (2017). Double ovarian stimulation during the follicular and luteal phase in women >/=38 years: a retrospective case-control study. Reprod Biomed Online.

[6] Peng Q, Cao X, Wang J (2019). Progestin-primed ovarian stimulation vs mild stimulation in women with advanced age above 40: a retrospective cohort study. Reprod Biol Endocrinol.

[7] Konig TE, van der Houwen LE, Overbeek A (2013). Recombinant LH supplementation to a standard GnRH antagonist protocol in women of 35 years or older undergoing IVF/ICSI: a randomized controlled multicentre study. Hum Reprod.

[8] Schmitz C, Bocca S, Beydoun H, Stadtmauer L, Oehninger S (2012). Does the degree of hypothalamic-pituitary-ovarian recovery after oral contraceptive pills affect outcomes of IVF/ICSI cycles receiving GnRH-antagonist adjuvant therapy in women over 35 years of age?. J Assist Reprod Genet.

[9] Sen A, Prizant H, Light A (2014). Androgens regulate ovarian follicular development by increasing follicle stimulating hormone receptor and microRNA-125b expression. Proc Natl Acad Sci U S A.

[10] Guo J, Zhang Q, Li Y (2014). Predictive value of androgens and multivariate model for poor ovarian response. Reprod Biomed Online.

[11] Dai K, Xu H, Ouyang N (2019). Correlation of human telomerase reverse transcriptase single nucleotide polymorphisms with in vitro fertilisation outcomes. J Assist Reprod Genet.

[12] Sunkara S, Pundir J, Khalaf Y (2011). Effect of androgen supplementation or modulation on ovarian stimulation outcome in poor responders: a meta-analysis. Reprod Biomed Online.

[13] Antonio R, Julio H, Jose L (2008). Use of letrozole in assisted reproduction: a systematic review and meta-analysis. Hum Reprod Update.

[14] Ferraretti AP, La Marca A, Fauser BC (2011). ESHRE consensus on the definition of ‘poor response’ to ovarian stimulation for in vitro fertilization: the Bologna criteria. Hum Reprod.

[15] Esteves SC, Roque M, Bedoschi GM, Conforti A, Humaidan P, Alviggi C (2018). Defining low prognosis patients undergoing assisted Reproductive Technology: POSEIDON Criteria-The why. Front Endocrinol (Lausanne).

[16] Gallot V, Berwanger da Silva A, Genro V, Grynberg M, Frydman N, Fanchin R (2012). Antral follicle responsiveness to follicle-stimulating hormone administration assessed by the follicular output RaTe (FORT) may predict in vitro fertilization-embryo transfer outcome. Hum Reprod (Oxford England).

[17] Huang MC, Tzeng SL, Lee CI (2018). GnRH agonist long protocol versus GnRH antagonist protocol for various aged patients with diminished ovarian reserve: a retrospective study. PLoS ONE.

[18] Chen Y, Qi Q, Xie Q, Yang Y, Xia Y, Zhou X (2018). Effect of Progestin-primed ovarian stimulation protocol on outcomes of aged infertile women who failed to get pregnant in the first IVF/ ICSI cycle: a self-controlled study. Curr Med Sci.

[19] Caldwell A, Edwards M, Desai R (2017). Neuroendocrine androgen action is a key extraovarian mediator in the development of polycystic ovary syndrome. Proc Natl Acad Sci U S A.

[20] Cornil CA (2018). On the role of brain aromatase in females: why are estrogens produced locally when they are available systemically?. J Comp Physiol A Neuroethol Sens Neural Behav Physiol.

[21] Wu X, Zhang Y, Xu S (2019). Loss of Gsdf leads to a dysregulation of Igf2bp3-mediated oocyte development in medaka. Gen Comp Endocrinol.

[22] Liu X, Xiao H, Jie M (2020). Amh regulate female folliculogenesis and fertility in a dose-dependent manner through Amhr2 in Nile tilapia. Mol Cell Endocrinol.

[23] McNairn A, Chuang C, Bloom J, Wallace M, Schimenti J (2019). Female-biased embryonic death from inflammation induced by genomic instability. Nature.

[24] Hen P, Norbert G, Aritro S (2014). Androgen actions in the ovary: balance is key. J Endocrinol.

[25] Oktay K, Buyuk E, Libertella N, Akar M, Rosenwaks Z (2005). Fertility preservation in breast cancer patients: a prospective controlled comparison of ovarian stimulation with tamoxifen and letrozole for embryo cryopreservation. J Clin Oncology: Official J Am Soc Clin Oncol.

