# Supplementary material for: The effect of letrozole overlapped with gonadotropin on IVF outcomes in women with DOR or aged over 40 years old with repeated cycles
Source: J Ovarian Res. 2023 Sep 18;16:193. doi: 10.1186/s13048-023-01273-4 (PMC10506294; doi:10.1186/s13048-023-01273-4)
Supplement: Supplementary file 1 — Supplementary Material 1: Table 1 Ovarian stimulation protocols used in previous cycles of the modified letrozole groupSupplementary [file 13048_2023_1273_MOESM1_ESM.doc]

**Supplementary Table 1** Ovarian stimulation protocols used in previous cycles of the modified letrozole group.

| Ovarian stimulation protocol | Number | Proportion |
| --- | --- | --- |
| GnRH agonist long protocol | 9 | 18% |
| Ultra-long GnRH agonist protocol | 1 | 2% |
| Shorting-acting GnRH agonist protocol | 8 | 16% |
| GnRH antagonist protocol | 19 | 38% |
| Mild stimulation protocol | 12 | 24% |
| Nature cycle | 1 | 2% |
