# Supplementary figures and images for: The effect of letrozole overlapped with gonadotropin on IVF outcomes in women with DOR or aged over 40 years old with repeated cycles
Source: J Ovarian Res. 2023 Sep 18;16:193. doi: 10.1186/s13048-023-01273-4 (PMC10506294; doi:10.1186/s13048-023-01273-4)

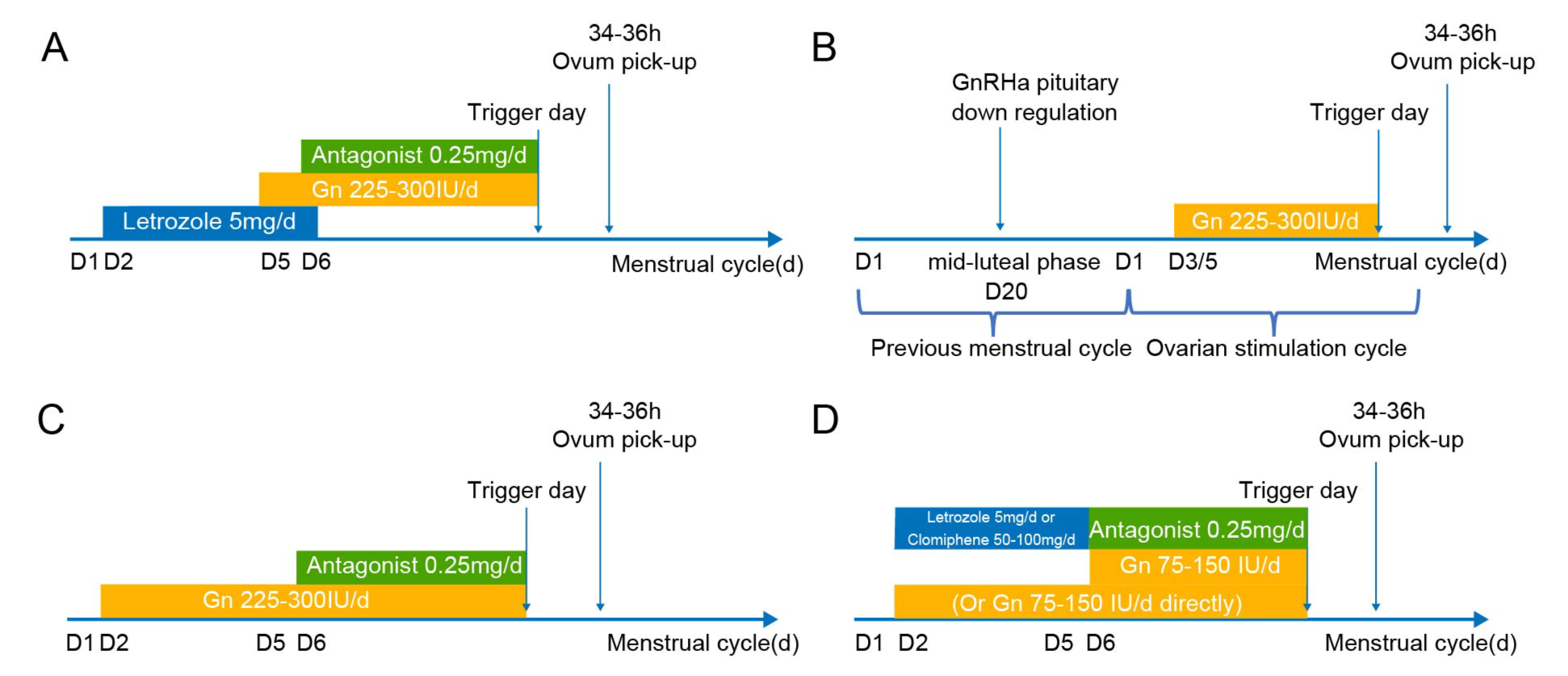

Supplement: Supplementary file 2 — Supplementary Material 2: Figure 1 Treatment scheme for four ovarian stimulation protocols. (A) Modified letrozole protocol. (B) GnRH agonist long protocol. (C) GnRH antagonist protocol. (D) Mild stimulation protocol [file 13048_2023_1273_MOESM2_ESM.tif]

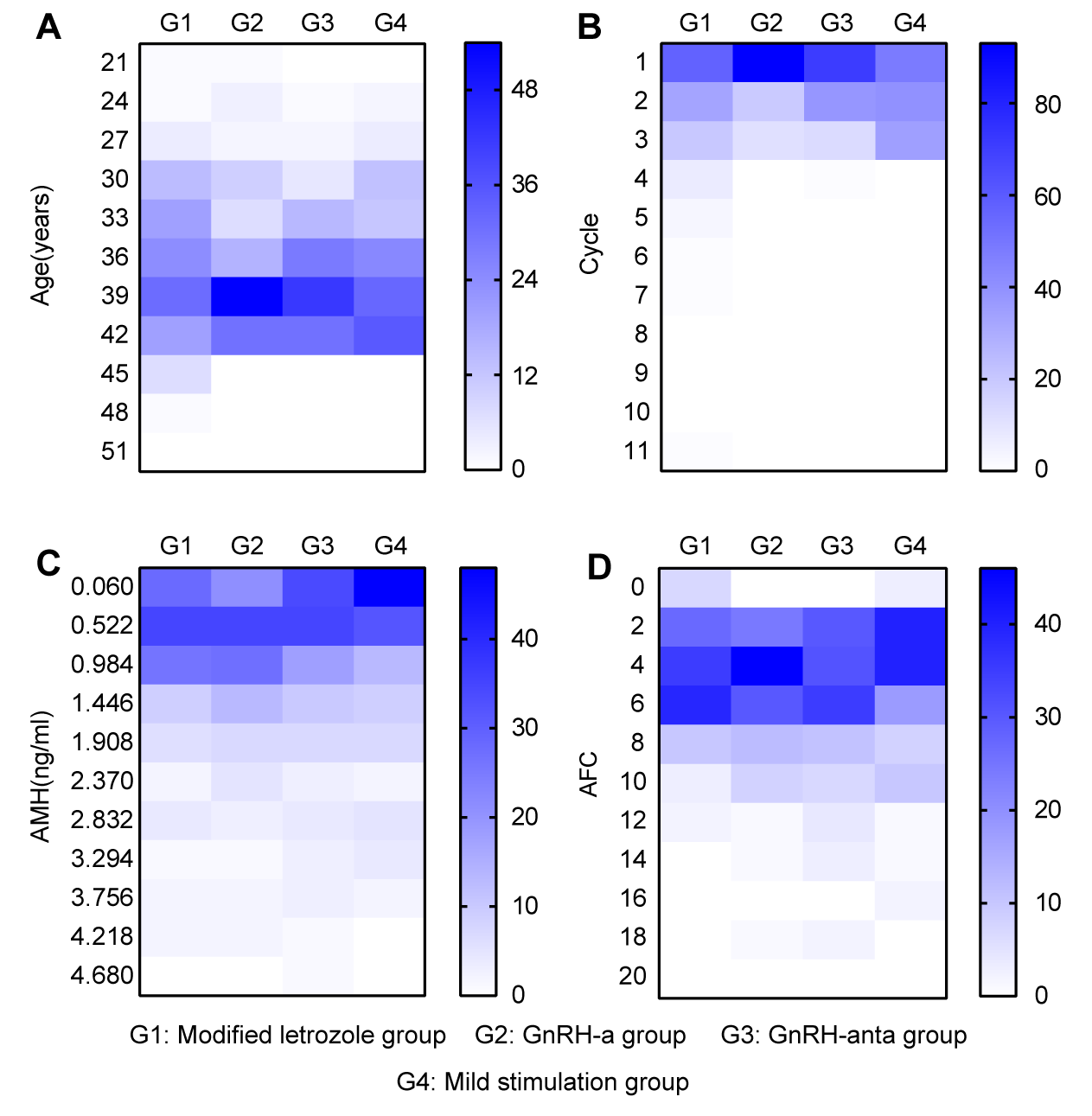

Supplement: Supplementary file 3 — Supplementary Material 3: Figure 2 (A-D) Distribution of age, cycle, serum AMH level, and antral follicle count in different groups. G1: Modified letrozole group. G2: GnRH agonist long group. G3: GnRH antagonist group. G4: Mild stimulation group [file 13048_2023_1273_MOESM3_ESM.tif]
